# Impact of termination of resuscitation for out-of-hospital cardiopulmonary arrest in Japan

**DOI:** 10.1186/cc12241

**Published:** 2013-03-19

**Authors:** T Fukuda, N Ohashi, M Gunshin, T Matsubara, S Nakajima, Y Kitsuta, N Yahagi

**Affiliations:** 1 Graduate School of Medicine, The University of Tokyo, Japan

## Introduction

What can one anticipate from the introduction of termination of resuscitation (TOR) for patients suffering out-of-hospital cardiopulmonary arrest (OHCA) in Japan? Irrespective of whether patients have made a living will requesting that medics do not attempt resuscitation, efforts are made to resuscitate over 90% of OHCA patients in Japan [1,2]; the number of people resuscitated exceed 120,000 every year. The 2010 American Heart Association (AHA) Guidelines for cardiopulmonary resuscitation (CPR) and emergency cardiovascular care (ECC) defined the criteria for TOR; this initiative may help reduce the number of unnecessary hospital transports by 40 to 60% and hold down medical costs [3].

## Methods

This was a single-center retrospective cohort study of patients who suffered OHCA and were transported to our hospital between April 2009 and March 2011. We investigated the patients' characteristics, whether they met the TOR criteria, and their outcome at the time of hospital discharge.

## Results

A total of 195 patients (mean age, 69 years), 67% of whom were male, were transported to our hospital after suffering OHCA. Cardiopulmonary arrest was witnessed in 52 cases (27%). The 2010 AHA Guidelines for CPR and ECC regarding the criteria for TOR were applied in 126 cases (65%), of whom 113 (90%) were dead on arrival, and 13 were successfully resuscitated and admitted. The outcomes for these 13 patients were as follows: 10 died in the hospital, two patients were discharged with a Glasgow Pittsburgh Cerebral Performance Category (CPC) score of 1, and one patient was transferred to another hospital with a CPC score of 3.

## Conclusion

In our study, 65% of the patients who were transported to the hospital after OHCA met the criteria for TOR. Outcomes for patients who met the TOR criteria were significantly worse than those who did not meet the criteria (2.4% vs. 14.5%, *P <*0.005). In Japan, efforts are made to resuscitate almost all individuals who suffer OHCA, but 75% of those patients die within a day. In light of the fact that even the medical cost for each of these patients who die within a day amounts to US$1,500 [4], the introduction of TOR will have a particularly strong impact in Japan.
